# Oral administration of marijuana produces alterations in serotonin 5-hydroxytryptamine receptor 3A gene (*HTR3A*) and electrolyte imbalances in brain of male Wistar rats

**DOI:** 10.22099/mbrc.2020.38601.1557

**Published:** 2021-03

**Authors:** Odunayo Anthonia Taiwo, Oluwatosin Adebisi Dosumu, Regina Ngozi Ugbaja, Solomon Oladapo Rotimi, Oluwafemi Paul Owolabi, Oluwafemi Adeleke Ojo

**Affiliations:** 1 Department of Biochemistry, Chrisland University, Owode, Abeokuta, Nigeria; 2 Department of Biochemistry, Federal University of Agriculture, Abeokuta, Nigeria; 3 Department of Biochemistry, Covenant University, Ota, Nigeria; 4 Department of Biochemistry, Landmark University, Omu-Aran, Nigeria

**Keywords:** Marijuana, Serotonin receptor gene, Gene expression, Neurotransmission

## Abstract

The gene expression of serotonin 5-hydroxytryptamine receptor 3A (receptor 3A:*HTR3A*) as well as the concentration of electrolytes in male Wistar rats after administration of graded doses of marijuana extract was investigated. Twelve groups (3 control and 9 test groups) of 6 animals each were daily exposed to 12.5, 25 and 50 mg/kg b.w doses of petroleum ether extract of marijuana for 4, 8 and 12 weeks. The expressions of the gene were obtained using reverse transcriptase-polymerase chain reaction (RT-PCR) while electrolytes concentrations were determined. An upregulation of over 90% was observed in the expression of *HTR3A* after exposure to the highest dose throughout the exposure period. There was significant increase in the plasma potassium concentration at all doses while there was a decrease in the brain only at 50 mg/kg dose throughout the exposure period. Sodium concentration in the brain was not affected by the doses over the period of exposure but plasma concentration decreased significantly. All the doses of marijuana extract significantly increased calcium concentration in the brain after prolonged exposure but the plasma concentration remained unchanged. This suggests that different doses of marijuana extract alter the expression of serotonin receptor and electrolyte concentrations over a period of time with possible neurological consequences.

## INTRODUCTION

Serotonin, otherwise called 5-hydroxytryptamine (5-HT), refers to a chemical messenger (neurotransmitter or neuromodulator) that is passed between nerve cells, crossing the synapse and received by a specific post synaptic receptor. It has been linked to almost all known human functions physiologically and behaviorally. It affects aggressive tendencies, desire for food, memory and cognition, vomiting, endocrine and gastrointestinal functions, motor and sensory coordination’s, neurotrophism, perception, sexual urges, sleep, and vascular functions [1].

There are about 14 serotonin receptor subtypes with multiple transduction mechanisms. The serotonin receptor 3A (5-HT3) is a Cys-loop ligand-gated ion channels (LGICs) receptor which contrasts from all other receptors in structure and function [2]. According to Bruss et al. [3], the serotonin receptor 3A gene is found on chromosome 11 with 7 exons and spans about 14.5 kb. 

The 5-HT3 receptor consists of a central ion conducting pore surrounded by five (5) subunits. The central pore allows free flow of sodium (Na), potassium (K), and calcium (Ca2+) ions. When serotonin binds to the receptor, the channel is opened, this results to an excitatory response in neurons. Sodium and potassium ions are responsible for the inward movement of the activating current [4], however, the permeability of 5-HT3 receptors to anions is minimal. The receptor is expressed all around the nervous systems and it is associated with diverse physiological roles [5]. Within the cells, postsynaptic 5-HT3 receptors serves as a link in rodent neocortical interneurons, hippocampus and in ferret visual cortex for rapid excitatory synaptic transmission [6]. Their presence on presynaptic nerve terminals and involvement in chemotherapy- and radiotherapy-triggered vomiting, has led to the advancement of specific 5-HT3 receptor antagonists to suppress these reactions and has raised significant awareness in the drug industry [7].

The electrolytes, majorly sodium and potassium are responsible for producing action potentials in neurons and ultimately for generating thoughts and actions. The heart, muscle and nerve cells employ electrolytes to keep up voltages over their cell bio-membranes and to move electrical stimuli to different cells [8]. All membranes are charged electrically because of the concentration of ions existing in the extracellular and intracellular space. Electrolytes controls the nerve and muscle function, hydration of the body, blood pH, blood pressure, oxygen delivery and repair of damaged tissue. Their concentrations within are kept under strict control by different mechanisms, controlled by hormonal actions and the kidneys [9]. 

Marijuana is made up of leaves, flowers, stems and seeds from the hemp plant, Cannabis sativa. Tetrahydrocannabinol (THC) over activates certain receptors of the brain cells, resulting in either physical and/or mental effects, such as: difficulty in body movement, thinking and analyzing, impaired memory and learning, possible damage to a fetus’ brain in pregnant subjects, hallucinating and paranoid feelings [10]. The time-course effect of Cannabis sativa on brain acetylcholinesterase (AChE) activity and expression of dopa decarboxylase Gene (DDC) was also reported by [11]. Its effect on neurotransmission through interaction with different electrolyte concentrations in the brain and gene expression is of particular interest in this study.

## MATERIALS AND METHODS

**Collection of Marijuana:** Marijuana was obtained from the National Drug Law Enforcement Agency (NDLEA). It was dried at 25°C and pulverized. It was soaked in petroleum ether for about 24 hours and filtered. The filtrate was concentrated using a rotary evaporator (RE300 DB) at 40oC. The concentrated extract was dissolved in olive oil at 50 mg/ml and kept in a dark bottle. The GC-MS analysis of the extract was done to determine and quantify the constituents of the plant extract. Out of thirteen (13) compounds identified, THC accounts for about 60.363% and this was the most abundant in the extract. Other constituents are: 9,12-Octadecadienoic acid (Z, Z), Cannabicoumaronone, 5H-Naphtho[2,3-c]carbazole, Morphinan-6-one, 8a-Methyl-5-methylene-3-(prop-1-en-2-yl), Ethyl Oleate-9- Tetradecenal, (Z)- Cyclopropaneoctanal, Linoelaidic acid, Cannabichromene, Dronabinol, 6-Methyl-2-phenyl-7-phenylmethylin dolizine, Cannabinol and Sterigmatocystin constituted less than 5% each in the extract.

**Animals:** A total of seventy-two male Wistar rats with weighing between 100 ± 8.66 g were used for this research. They were purchased from Anatomy Department, College of Veterinary medicine, Federal university of Agriculture (FUNAAB), Alabata, Abeokuta, Ogun State. The rats were kept in clean plastic cages under standard 12-h light and dark cycles and could access food and clean water ad libitum. The rats were acclimatized for two weeks before the start of the research and were taken care of according to the declaration of Helsinki.

**Treatment method and tissue harvesting: **Experimental animals were divided into twelve groups (3 control and 9 test groups) of six animals each. The test groups were administered 12.5, 25 and 50 mg/kg body weight marijuana extract for 4, 8 and 12 weeks respectively. Each group of animals was anesthetize with diethyl ether and euthanized by exsanguinations by cardiac puncture at the end of the exposure time. Blood was obtained by cardiac puncture into lithium-heparin tubes. The whole blood was centrifuged immediately for 10 minutes at 4000 rpm to obtain the plasma. The brain was removed and a portion placed in RNAlater®for ribonucleic acid (RNA) extraction analysis. Another portion of the brain (0.1 g) was homogenized in 0.9 ml of 0.1 M Tris buffer (pH 7.4) for electrolyte analysis. The experimental procedure was approved by the departmental board of the Department of Biochemistry, Federal University of Agriculture Abeokuta, Nigeria.

**RNA extraction and gene expression of HTR3A: **RNA was extracted from the brain with Aidlab spin column RNA extraction kit as per the maker's guidelines. A NanoDrop® 2000 spectrophotometer (Thermo Scientific) was utilized to quantify the total RNA yield providing the 260nm/280nm quotient of the sample to determine the concentration and purity. The degrees of expression of serotonin receptor 3A genes were determined in the brain extract by reverse transcriptase-polymerase chain reaction (RT-PCR). This technique combines the first-strand complementary DNA (cDNA) synthesis with polymerase chain reaction (PCR) in a similar cylinder to improve reaction response and decrease the chance of contagion. The RT-PCR was done with 500 ng RNA format utilizing TranGenEasyScript one-step RT-PCR pack as per producer's directions. The RNA samples were exposed to the accompanying conditions: cDNA synthesis at 45ºC for 30 min, denaturation at 94ºC for 5 min, 40 cycles of (94 ºC for 30 secs., annealing at 52ºC for 30 secs., extension at 72ºC for 1 min.) using gene specific primer (Table 1) and final extension at 72ºC for 10 min. The process lasted for some hours before an agarose gel electrophoresis was carried out.

Amplifications were analyzed in C1000 Touch™ Thermal Cycler (Bio-Rad Laboratories, Hercules, CA). The amplicons were envisioned on 1% agarose gel in 1X Tris Borate ethylenediaminetetraacetic acid (EDTA) buffer utilizing UVP BioDoc-It- ™ Imaging system (Upland, CA, USA). Data obtained were expressed as gene expression using image J software (Abràmoff et al., 2004).

**Table 1:** Sequences of gene-specific primer

**Gene Specific Primers**	**Sequence (5`-3`)**	**Template**
HTR3A	Forward: GACTGCTCAGCCATGGGAAA	NM_024394.2
	Reverse: AGCCATGATAGCGAAGTCGG	
β-Actin	Forward: GTCAGGTCATCACTATCGGCAAT	NM_031144.3
	Reverse: AGAGGTCTTTACGGATGTCAACGT	

**Determination of concentration of electrolytes: **The concentration of all the four electrolytes (Na^+^, K^+^, Ca^2+^, Mg^2+^) in the plasma and brain was resolved spectrophotometrically as per the strategy portrayed in Randox diagnostic kit manual.

**Data analyses: **Data were expressed as Mean ± SEM of six replicates in each group. One-way analysis of variance (ANOVA) was done to test for level of homogeneity at p < 0.05 while Tukey multiple range test was utilized to separate the heterogeneous groups. 

## RESULTS

**Figure 1:**
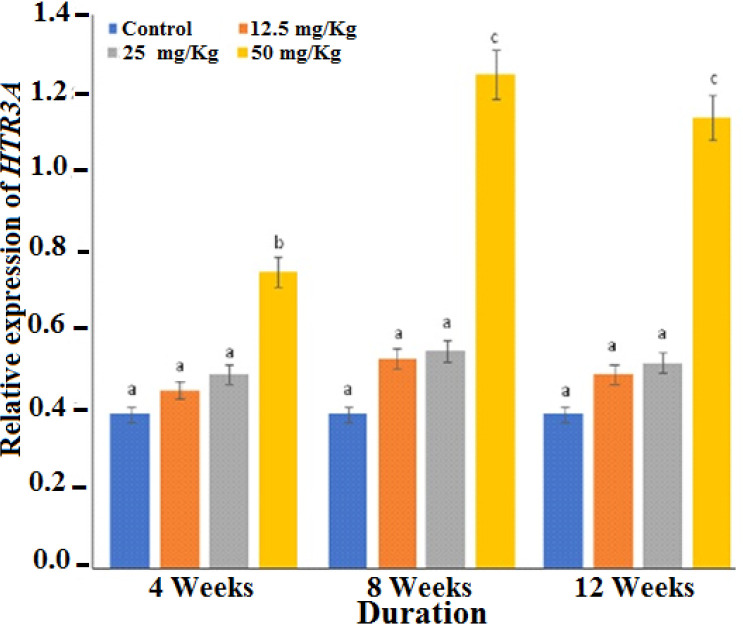
Expressions of Serotonin receptor 3A gene (HTR3A) in the brain

There was no considerable difference (p>0.05) in the relative expression of HTR3A after administration of 12.5 mg/kg and 25 mg/kg doses, however, 50 mg/kg dose led to a substantial increase (p < 0.05) throughout the period of Marijuana exposure (Fig. 1).

**Figure 2:**
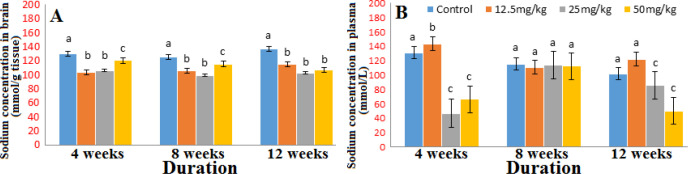
Concentration of sodium in brain (A) and plasma (B) after 4, 8 and 12 weeks’ exposure to graded doses of Marijuana. n=6; same alphabets shows no significant difference

**Figure 3:**
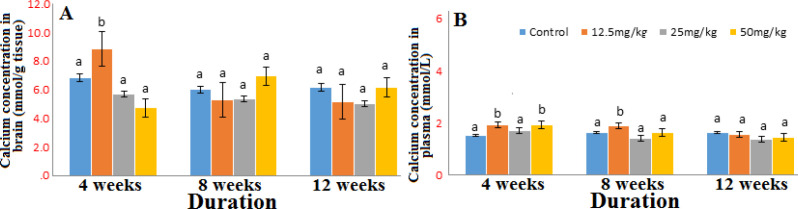
Concentration of calcium in brain (A) and plasma (B) after 4, 8 and 12 weeks’ exposure to graded doses of Marijuana. n=6; same alphabets shows no significant difference

**Figure 4:**
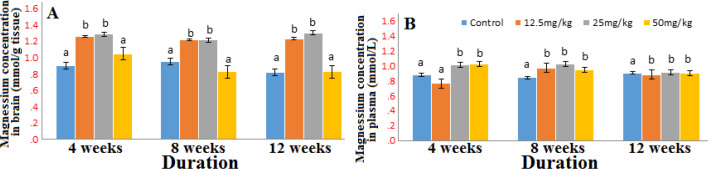
Concentration of magnesium in the brain (A) and plasma (B) after 4, 8 and 12 weeks’ exposure to graded doses of Marijuana. n=6; same alphabets shows no significant difference

The concentrations of sodium in the brain and in the plasma (Fig. 2) were reduced significantly at all doses throughout the duration of exposure. The brain potassium concentration was reduced (Fig. S1) however, there was an increase in the plasma concentration at all doses (Fig. S2). There were slight variations in the concentrations of calcium and magnesium in the brain and plasma producing increases at some doses and decreases at others in the course of the exposure period (Fig. 3-4).

## DISCUSSION

5-Hydroxytryptamine Receptor 3A (HTR3A) is essential in transmission of nerve impulses across cell membranes, stimulating vomiting reflexes, enhancing cognition in rats, which could likely suggest their potential use as memory enhancers, implicated in various disorders, neurological diseases and human breast cancer cell line (MCF-7 [12-15].

In this study, high doses of marijuana increased the expression of *HTR3A*. Low doses however, decreased the expression of the gene, this may be responsible for the suggested use of cannabis at low doses for the treatment of vomiting and nausea [7] and to also improve eating in HIV patients.

Controlling the distribution of fluid and maintaining acid-base equilibrium in the intra and extracellular space, are basic functions of electrolytes in the body. Result obtained from this study revealed that exposure to high doses of marijuana (25 mg/kg and 50 mg/kg) led to a decrease in potassium concentration in the brain but an increase in the plasma at all doses while the plasma and brain sodium concentration reduced i.e hypokalemia and hyponatremia [16, 17] respectively. The effects observed above corroborates the work of Osadolor and Emokpae, [18] and Hubbard *et al*, [19] who reported that marijuana smoking decreases potassium (K^+^) levels in smokers. It is also in line with the reasoning of some researchers that some drugs, loss of salt and water would lead to a decrease in sodium levels. 

Normal potassium levels are essential to the heart and brain function. Hypokalemia can hinder electrical signals that are needed by the brain and consequently lead to misperception, slow opinions or the incapability to initiate activities and brain fog [20]. 

High dosage of marijuana may lead to hypokalemia in the brain which may be linked to either the inhibition of adenylate cyclase by cannabinoids; an active ingredient in marijuana that affects potassium channel function to bring inhibition of synaptic transmission [21] or Tachycardia i.e. heart rate more than 100 beats per minute which is one of the main peripheral effect of marijuana intake [18]. There could probably have been a redistribution of ions due to an efflux of potassium ions from the brain to the plasma. Cannabidiol has been implicated in altering the membrane fluidity and also to directly bind to sodium channels which could be the mechanism involved in the hyponatremia observed in this study [22] consequent mild or severe symptoms [23,24].

There seems to be very mild effect of marijuana on magnesium concentration in both the plasma and brain. The increase in the brain calcium and magnesium concentration of the marijuana exposed group as observed in this study might be an indicator that marijuana promotes influx of Ca^2+^ and Mg^2+^ in the brain. It is possible that the marijuana like some toxicants might have altered the structure of the membrane of endoplasmic reticulum (ER) and mitochondria which stores calcium, the alteration might cause influx of calcium to the brain and in turn increase intracellular calcium level. Abam *et al*, [25] noted that calcium and magnesium homeostasis may be affected by toxicants and drugs either by enhancing their influx into or preventing their efflux from the cytoplasm. In addition, inhibiting the ion pumps or depleting the ion pumps of their driving forces may reduce the efflux of these ions [26, 27]. Reduced influx of calcium has been reported to decrease directly the activation of destructive pathways [28] therefore; increased influx may speed up the activation of destructive pathways. 

Although there were slight variations in the concentration of magnesium at different doses and duration of exposure, magnesium imbalance rarely occur, as the kidneys are very effective in regulating its concentration and symptoms are very mild or absent [29].

## Supplementary Materials

Supplement
