# Supplementary material for: Oral administration of marijuana produces alterations in serotonin 5-hydroxytryptamine receptor 3A gene (HTR3A) and electrolyte imbalances in brain of male Wistar rats
Source: Mol Biol Res Commun. 2021 Mar;10(1):5–11. doi: 10.22099/mbrc.2020.38601.1557 (PMC7936388; doi:10.22099/mbrc.2020.38601.1557)
Supplement: Supplement [file mbrc-10-5-s001.pdf]

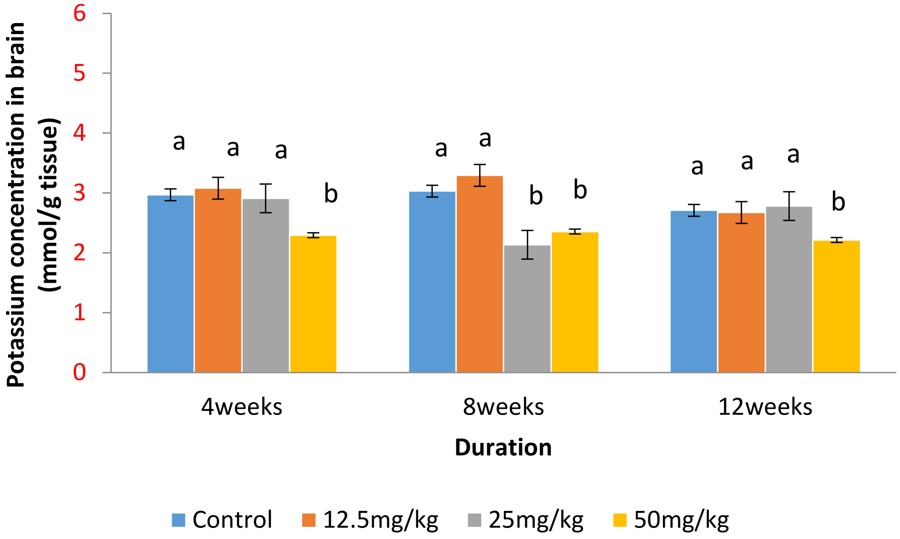

Figure S1: Concentration of potassium in the brain after 4, 8 and 12 weeks' exposure to graded doses of Marijuana

Values are expressed as mean ± standard error (SE); n = 6
Bar with the same alphabets are not significant difference at $p > 0.05$
Bar with different alphabets are significant difference at $p < 0.05$

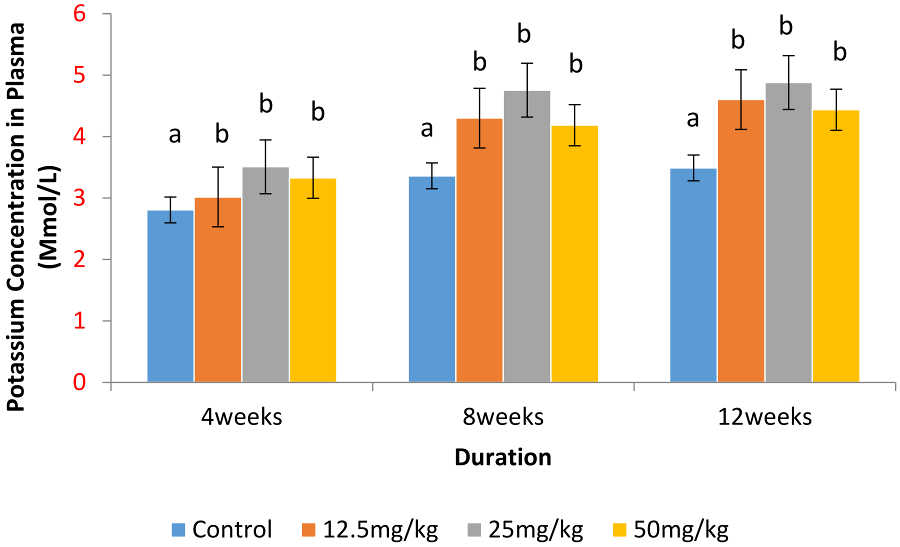

Figure S2: Concentration of potassium in the Plasma after 4, 8 and 12 weeks' exposure to graded doses of Marijuana

Values are expressed as mean ± standard error (SE); n = 6
Bar with the same alphabets are not significant difference at $p > 0.05$
Bar with different alphabets are significant difference at $p < 0.05$
